# Characteristics and outcome of patients with small bowel adenocarcinoma (SBA)

**DOI:** 10.1007/s00432-022-04344-z

**Published:** 2022-09-26

**Authors:** Andreas Teufel, Nadja M. Meindl-Beinker, Pauline Hösel, Michael Gerken, Ana Roig, Matthias P. Ebert, Wolfgang Herr, Alexander Scheiter, Armin Pauer, Hans J. Schlitt, Monika Klinkhammer-Schalke

**Affiliations:** 1 Division of Hepatology, Division of Clinical Bioinformatics, Department of Medicine II, Medical Faculty Mannheim, Heidelberg University, Theodor-Kutzer-Ufer 1-3, 68167 Mannheim, Germany; 2 Clinical Cooperation Unit Healthy Metabolism, Center for Preventive Medicine and Digital Health, Medical Faculty Mannheim, Heidelberg University, Mannheim, Germany; 3 Department of Medicine II, Medical Faculty Mannheim, Heidelberg University, Mannheim, Germany; 4 DKFZ-Hector Cancer Institute at the University Medical Center, Mannheim, Germany; 5 Mannheim Institute for Innate Immunoscience (MI3), Medical Faculty Mannheim, Heidelberg University, Mannheim, Germany; 6 Bavarian Cancer Registry, Regional Center Regensburg, Bavarian Health and Food Safety Authority, Regensburg, Germany; 7 Department of Internal Medicine III, University Medical Center Regensburg, Regensburg, Germany; 8 Department of Pathology, University of Regensburg, Regensburg, Germany; 9 Regensburg Tumor Center, Institute for Quality Assurance and Health Services Research at the University of Regensburg, Regensburg, Germany; 10 Department of Surgery, University Medical Center Regensburg, Regensburg, Germany

**Keywords:** Small bowel, Adenocarcinoma, Chemothherapy, Survival, Register, Tumo center

## Abstract

**Background:**

Small bowel adenocarcinoma (SBA) remains a rare malignancy accounting for less than 5% of all the gastrointestinal tract cancers. However, only limited data and expert guidelines are available for this entity. As a result, treatment concepts are predominantly derived from colorectal cancer.

**Methods:**

To substantiate data on the course of disease, diagnosis and treatment of SBA, we performed a population-based analysis from a Bavarian population of 2.2 million people.

**Results:**

We identified 223 patients with SBA. Mean age at diagnosis was 67.8 years and patients were diagnosed rather late (34.5% UICC stage IV). Largest proportion of these patients were diagnosed with adenocarcinoma of the duodenum (132 patients, 59.2%) and most patients were diagnosed with late stage cancer, stage IV (70 patients, 31.4%). With respect to treatment, most patients underwent primary surgery (187 patients, 84.6%). Systemic therapy seemed to have an impact in UICC stage IV patients but not in UICC stage IIB or III. The 5-year survival rate was 29.0%. This was significantly less compared to colon cancer in the same cohort, which was 50.0%. Furthermore, median survival of patients with small bowel cancer was only 2.0 years (95% CI 1.4–2.5) compared to 4.9 years (95% CI 4.8–5.1) of patients with colon cancer.

**Conclusion:**

SBA showed a distinct epidemiology compared to colon cancer. Thus, data acquisition particularly on systemic treatment are paramount, with the objective to complement the available guidelines.

**Supplementary Information:**

The online version contains supplementary material available at 10.1007/s00432-022-04344-z.

## Introduction

Small bowel cancer disease remains a rare malignancy accounting for less than 5% of all gastrointestinal tract cancers (Hatzaras et al. 2007); (Sakae et al. 2017). Between 55 and 82% of the cases are located in the duodenum, followed by the jejunum. Only 7–17% of cases occur in the ileum (Aparicio et al. 2014). According to epidemiological data, small bowel disease incidence has been rising in many countries from the 1900’s until today (Aparicio et al. 2014). Specifically, data from the EUROCARE indicate around 3.600 new SBA cases per year in Europe (Sakae et al. 2017). Among the distinguished subtypes of small bowel cancer adenocarcinoma, carcinoma, sarcoma, gastrointestinal stromal tumor or lymphoma cancer, small bowel adenocarcinoma (SBA) predominates with 40% incidence (Sakae et al. 2017); (Farhat et al. 2008) and has been studied more in depth than other subtypes.

However, as a consequence of the low incidence of small bowel cancer, there is a lack of extensive clinical, aetiological and pathological information and it remains challenging to diagnose, treat, understand and prevent the disease. (Aparicio et al. 2014); (Farhat et al. 2008). In addition, clinical presentation of small bowel malignancies tends to be delayed as unspecific symptoms jeopardise early detection of the disease.

As large-scale studies on small bowel cancers are widely lacking, most cancers are treated following recommendations for large bowel tumors. Thus, depending on tumor stage and patient’s condition, therapeutic options generally include wide segmental surgical resection of the tumor and chemotherapy (Aparicio et al. 2014); (Hirao et al. 2017). However, more clinical data and patient follow-up are required to further assess the effects of both treatments and perhaps suggest new or combinatory therapeutic options which may improve survival. So far, median overall survival of small bowel cancer patients is approximately 5 years (Hatzaras et al. 2007); (Aparicio et al. 2014); (Sakae et al. 2017) as a consequence of delayed diagnosis and unspecific symptoms.

To further substantiate evidence and knowledge on small bowel tumors, we analysed the overall survival of 223 patients from the Regensburg Tumor Centre suffering from SBA and compared it to large bowel tumors.

## Patients and methods

Retrospective analysis of clinical data was performed based on the population-based cancer registry at the Regensburg Tumor Center in Eastern Bavaria, Germany. Epidemiological and clinical data on small bowel cancers were investigated in a cohort of patients diagnosed between 2002 and 2020.

### Ethics approval

The study design was reviewed and approved by the Ethical Review Board of the University of Regensburg, Germany (approval no. 15–170-0000). All procedures performed in this study were in accordance with the ethical standards of the institutional and national research committee and with the 1964 Helsinki Declaration and its later amendments. This article does not contain any studies with human participants or animals performed by any of the authors.

### Data collection

The cancer registry of the Regensburg Tumor Center records epidemiological and clinical data from all patients with malignancies diagnosed and treated by clinicians in Upper Palatinate and in Eastern Bavaria. The region covered by the registry consists of about 2.2 million inhabitants. More than 1,000 practicing physicians, the University Hospital of Regensburg, and 53 regional hospitals are involved in the area-wide, population-based, cross-sector documentation of cancer patients. The registry receives medical information from all regional pathologists and clinicians at the time of diagnosis, treatment, and during follow-up. Physicians may enter the data in case forms, use computer-assisted tumor documentation, or send medical reports to the registry. At the office of the registry the data are extracted, recorded, and fed into a central database by suitably trained personnel. The patients’ survival status and disease recurrence are obtained from clinical reports, death certificates issued by the local public health departments and the registration offices of the respective residential districts. Data are processed and secured according to the Bavarian Law of Cancer Registries. According to the estimates of the German Robert Koch-Institute (RKI), the Regensburg Tumor Center includes more than 90% of the estimated number of cancer cases. Thus, the data were comprehensive and selection bias was largely excluded.

**Fig. 1:**
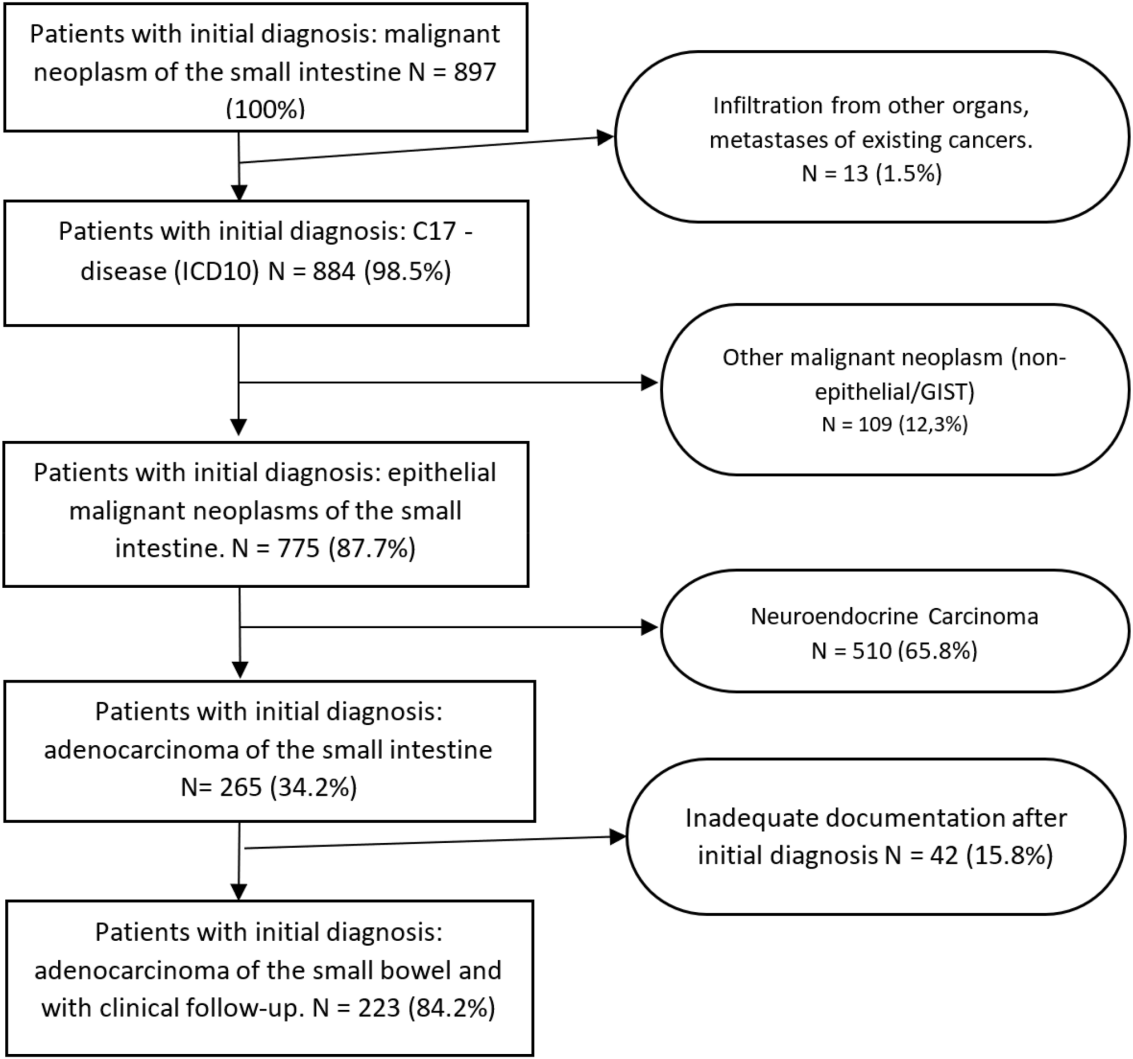
Flowchart for inclusion and exclusion of patients in the analysis

The baseline cohort of the present study consisted of patients with the ICD-10-GM (http://www.dimdi.de/static/de/klassi/icd-10-gm/index.htm) diagnosis “C17 adenocarcinoma of the small bowel, duodenum, jejunum, and ileum”. Patients with histologically confirmed small bowel cancer documented in the cancer registry between 2002 and 2020 were included in the study. Patients with neuroendocrine carcinoma and with inadequate documentation were excluded (Fig. 1).

Patients with colon cancer were selected based on the ICD-10 code “C18 colon “ between 2000 and 2018. Rectal carcinoma patients were not included.

### Statistical analysis

Continuous data are described as means, median, minimum, maximum values and standard deviation. Categorical data are expressed as absolute frequencies and percentages. Patient characteristics were compared with t-tests for normally distributed continuous data, tested by Kolmogorov–Smirnov test and Chi-square tests for categorical variables. Life status for estimating overall survival rates was determined from clinical reports, death certificates, and registration offices. The follow-up period and survival times were right censored using December 31, 2020 as the cut-off date. Overall survival rates were estimated by Kaplan–Meier method, and univariable and multivariable Cox-regression analyses. Differences were tested for statistical significance by the two-sided Log-Rank test. To determine the influence of further co-variables on overall survival and second cancer, we performed multivariable regression analysis using Cox proportional hazard models. In multivariable analysis, the hazard ratio (HR) was adjusted for the co-variables of age at diagnosis, sex, comorbidity according to Charlson Comorbidity Index, tumor localization, tumor size, nodal status, primary distant metastases, histopathological grading, lymph vessel and vein invasion. Hazard ratios and corresponding 95% confidence intervals (CI) were estimated, and considered statistically significant when the CI excluded 1.0. The proportional hazard assumption was tested by comparison in log-minus-log (LNL) plots. A p-value of < 0.05 was considered to be statistically significant. All analyses were performed using IBM SPSS Statistics, version 25.0 (IBM SPSS Statistics, Armonk, NY, USA).

## Results

### Demographic and tumor characteristics of patients with small bowel cancer

Overall, we identified 223 patients with small bowel cancers, 128 were male and 95 were female. No significant differences between male and female patients were detected in terms of age at diagnosis, comorbidity index, localization (ICD10 code), stage UICC, grading, lymphatic and venous invasion, surgical resection and systemic therapy between both groups (Table 1). Gender disparities were excluded.

**Table 1:** Distribution of tumor characteristics (age at diagnosis, Charlson comorbidity index, diagnosis by ICD10 code, stage UICC, grading, lymphatic vessel invasion, venous invasion) and therapy (primary surgery (yes/no), primary systemic therapy (yes/no)) in the total collective by gender with p-value (chi-square test)

		Sex						
		Female		Male		Total		Chi-square
		*N*^1^	(%)	*N*1	(%)	*N*1	(%)	*p*
Diagnostic age	30.0 – 39.9	3	3.2	4	3.1	7	3.1	0.771
	40.0 – 49.9	7	7.4	5	3.9	12	5.4	
	50.0 – 59.9	15	15.8	23	18.0	38	17.0	
	60.0 – 69.9	23	24.2	34	26.6	57	25.6	
	70.0 – 79.9	29	30.5	44	34.4	73	32.7	
	80.0 – 89.9	18	18.9	18	14.1	36	16,1	
Charlson-Comorbidity-Index	0	57	60.0	63	49.2	120	53.8	0.374
	1	22	23.2	34	26.6	56	25.1	
	2	7	7.4	11	8.6	18	8.1	
	3 +	9	9.5	20	15.6	29	13.0	
Localization ICD10 Code	C17.0 (Duodenum)	55	57.9	77	60.2	132	59.2	0.114
	C17.1 (Jejunum)	20	21.1	33	25.8	53	23.8	
	C17.2 (Ileum)	17	17.9	10	7.8	27	12.1	
	C17.8 (Several subareas)	3	3.2	5	3.9	8	3.6	
	C17.9 (unknown)	0	0.0	3	2.3	3	1.3	
Stage UICC	I	7	7.4	6	4.7	13	5.8	0.623
	IIA	14	14.7	16	12.5	30	13.5	
	IIB	9	9.5	10	7.8	19	8.5	
	IIIA	11	11.6	21	16.4	32	14.3	
	IIIB	6	6.3	11	8.6	17	7.6	
	IV	34	35.8	36	28.1	70	31.4	
	Unknown	14	14.7	28	21.9	42	18.3	
Grading	G1/G2	58	61.1	70	54.7	128	57.4	0.452
	G3/G4	31	32.6	52	40.6	83	37.2	
	GX/ns^2^	6	6.3	6	4.7	12	5.4	
Lymphatic vessel invasion	L0	32	33.7	43	33.6	75	33.6	0.788
	L1	29	30.5	44	34.4	73	32.7	
	LX/ns2	34	35.8	41	32.0	75	33.6	
Venous invasion	V0	51	53.7	66	51.6	117	52.5	0.666
	V1/V2	7	7.4	14	10.9	21	9.4	
	VX/ns2	37	38.9	48	37.5	85	38.1	
Operation	No	13	13.7	14	11.1	27	12.2	0.641
	Yes	80	84.2	107	84.9	187	84.6	
	Unknown	2	2.1	5	4.0	7	3.2	
Systemic Therapy	No	43	45.3	56	43.8	99	44.4	0.332
	Yes	31	32.6	54	42.2	85	38.1	
	Planned	9	9.5	7	5.5	16	7.2	
	Unknown	12	12.6	11	8.6	23	10.3	
Total		95	100.0	128	100.0	223	100.0	

^1^Number of patients

^2^NS not specified

Investigating subgroups, most patients were diagnosed at late-age between 70.0 and 79.9 years old. Those were in total 73 patients (32.7%), 29 (30.5%) female and 44 (34.4%) male patients. Only very few patients were diagnosed at an early age of less than 50 years (19 patients, 8.5%). Mean age at diagnosis was 67.8 years (median 69.4, range 32.4–89.4) and almost identical in women and men (*p* = 0.907). By far, the largest proportion of these patients were diagnosed with adenocarcinoma of the duodenum (132 patients, 59.2%). Again, this finding was similar among men and women. Most patients were diagnosed with late stage cancer, stage IV (70 patients, 31.4%). Stage distribution was comparable in patients with duodenal and non-duodenal cancer location, especially the portion of stage IV was almost similar in duodenal cancer patients with 31.1%. In the stage IV cohort half of the patients exhibited peritoneal metastases, 47.1% showed metastases in the liver, 14.3% in the lung, 5.7% in ovaries, 4.3% each in the mediastinum and bone, 1.4% each in colon and suprarenal gland.

Only 13 patients (5.8%) were diagnosed with early small bowel cancer, stage I (duodenal cancer 6.8%). The majority of tumors exhibited a favourable G1/G2 grading (128 patients, 57.4%).

With respect to treatment, most patients underwent primary surgery (187 patients, 84.6%). Surgery procedures were distributed as follows: 49 (26.2%) Whipple-operations, 14 (7.5%) gastroenterostomies, 19 (10.2%) partial duodenal resections, 39 (20.9%) partial non-duodenal resections, 7 (3.7%) duodenal segment resections, 43 (23.0%) non-duodenal segment resections, and 16 (8.5%) other types of surgery. Among 108 patients with primary surgery in stages I–III, the rate of local R0-resections was 93.5%.

As for resection of SBA metastases: 8 patients underwent resection of liver metastases, 3 patients had gynecological surgery. 8 patients had additional resection of the colon, mostly right hemicolectomy or resection of colon ascendens and coecum. Pancreas resections were performed in 8 patients.

Only a minority of patients received systemic therapy (85 patients, 38.1%).

### Demographic and tumor characteristics of patients with colon cancer

For comparative purposes, we analyzed key clinical data of 11.966 colon carcinoma patients in the same population. In the colon cancer collective the proportion of men was 57.4%, mean age at diagnosis was 70.1 years, median age 71.5 years (range 11.8–101.6). The distribution of UICC stage was as follows: 20.1% stage I, 28.4% stage II, 24.0% stage III, and 27.4% stage IV. Systemic treatment according to German guidelines consisted mostly in adjuvant chemotherapy in patients with affected lymph nodes, UICC stage III and large T4 tumors whereas palliative or additive chemotherapy is applied in patients with primary metastases UICC IV or positive residual tumor.

### Overall survival of patients with small bowel adenocarcinoma and comparison with colon carcinoma

**Fig. 2:**
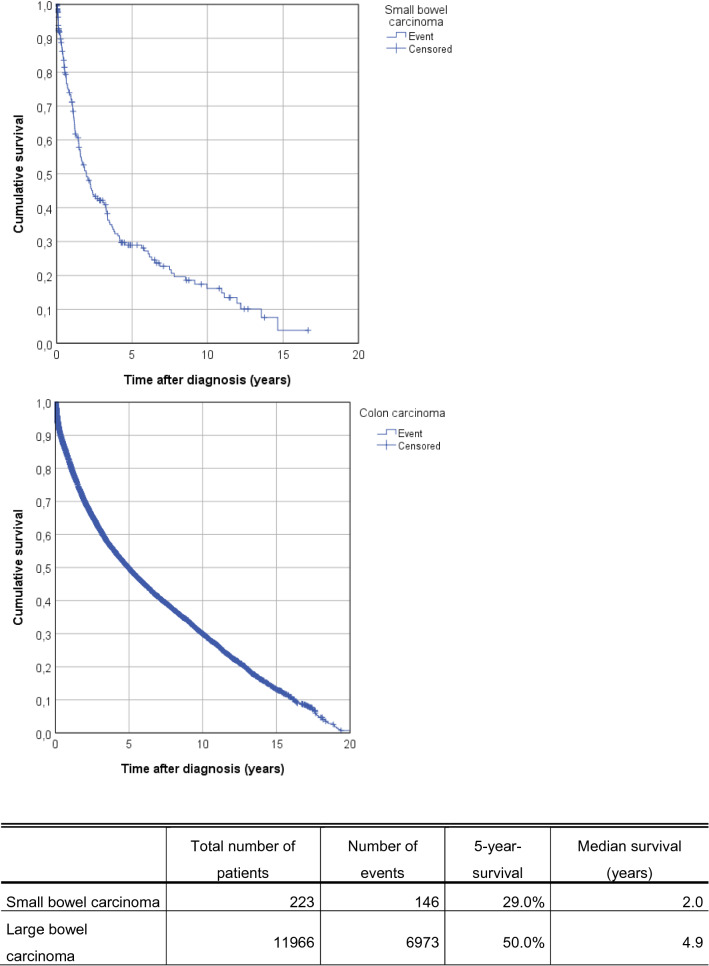
Comparison of the 5-year survival rate and the median survival between patients with small intestine and colon carcinomas

Mean follow-up was 7.3 years (median 6.5). Overall survival of the collective over the course of 5 years is presented in Fig. 2a. The 5-year survival rate was 29.0%. This is significantly less compared to large bowel tumors, which was 50.0%. Furthermore, median survival of patients with small bowel cancer was only 2.0 years (95% CI 1.4 – 2.5) compared to 4.9 years (95% CI 4.8 – 5.1) of patients with colon cancer.

### Later diagnosis is associated with worse prognosis

As most patients presented with late stage (stage IV) cancers, we next investigated whether this had an impact on the patient’s survival probability. We clearly observed that higher tumor stages were associated with a worse prognosis (suppl. Figure 1). While 71.1% of individuals diagnosed in stage I survived 5 years after diagnosis, only 4.3% of patients diagnosed at stage IV survived the same time period. As depicted in suppl. table 1, clearly there were significant statistical differences in survival between patients diagnosed at stage I with those at stages IIIB and IV individuals (*p* = 0.024 and *p* < 0.001, respectively).

### Systemic therapy in patients with small bowel cancer

As shown in Table 2 and in line with current treatment guidelines, patients with higher tumor stages at initial diagnosis were more likely to be treated with or planned for receiving primary systemic therapy. While 64.3% of patients diagnosed at stage IV (*n* = 45), only 7.7% (*n* = 1) patient diagnosed in stage I received systemic therapy.

**Table 2:** Distribution of tumor characteristics (gender, age at diagnosis, Charlson comorbidity index, diagnosis by ICD10 code, tumor size T, lymph node involvement N, grading, lymphatic vessel invasion, venous invasion) of stage IV patients at initial diagnosis after primary systemic therapy

		Primary systemic therapy						
		No		Yes		Total		Chi-square
		*N*^3^	(%)	*N*6	(%)	*N*6	(%)	*p*
Gender	Female	6	42.9	19	43.2	25	43.1	0.983
	Male	8	57.1	25	56.8	33	56.9	
Diagnosis age	30.0 – 39.9			3	6.8	3	5.2	0.115
	40.0 – 49.9			3	6.8	3	5.2	
	50.0 – 59.9	2	14.3	6	13.6	8	13.8	
	60.0 – 69.9	3	21.4	17	38.6	20	34.5	
	70.0 – 79.9	6	42.9	14	31.8	20	34.5	
	80.0 – 89.9	3	21.4	1	2.3	4	6.9	
Charlson-Comorbidity-Index	0	5	35.7	25	56.8	30	51.7	0.161
	1	4	28.6	13	29.5	17	29.3	
	2 +	5	35.7	6	13.6	11	19.0	
Localization ICD10 Code	C17.0 Duodenum	8	57.1	27	61.4	35	60.3	0.142
	C17.1 Jejunum	2	14.3	13	29.5	15	25.9	
	C17.2 Ileum	4	28.6	4	9.1	8	13.8	
Tumor size T	T1/T2			2	4.5	2	3.4	0.401
	T3	3	21.4	11	25.0	14	24.1	
	T4	4	28.6	19	43.2	23	39.7	
	TX/ns^4^	7	50.0	12	27.3	19	32.8	
Lymph node involvement N	N0	4	28.6	5	11.4	9	15.5	0.073
	N1	3	21.4	18	40.9	21	36.2	
	N2			8	18.2	8	13.8	
	NX/ns7	7	50.0	13	29.5	20	34.5	
Grading	G1/G2	6	42.9	21	47.7	27	46.6	0.802
	G3/G4	7	50.0	18	40.9	25	43.1	
	GX/ns^7^	1	7.1	5	11.4	6	10.3	
Lymphatic vessel invasion	L0	2	14.3	7	15.9	9	15.5	0.905
	L1	4	28.6	10	22.7	14	24.1	
	X/ns^7^	8	57.1	27	61.4	35	60.3	
Venous invasion	V0	4	28.6	13	29.5	17	29.3	0.997
	V1/2	1	7.1	3	6.8	4	6.9	
	X/ns^7^	9	64.3	28	63.6	37	63.8	
Total		14	100.0	44	100.0	58	100.0	

^3^Number of patients

^4^*Ns* not specified

To analyse the application of and the influence of primary systemic therapy on survival rates in more detail, we next analysed patients diagnosed at stage IIB and stage III (A and B) or patients with late-stage diagnosis (Stage IV), separately. In the cohort analysed in this study, *n* = 59 patients were diagnosed with stages IIB or III of which *N* = 28 (47.5%) individuals received primary systemic therapy (see suppl. Table 5). Besides stage (UICC) and lymph node involvement (*N*) (*p* = 0.013 and *p* = 0.013, respectively), there were no significant differences between the groups receiving systemic therapy or not with regard to sex, age at diagnosis, Charlson co-morbidity index, localization by ICD10 code, tumor size T, grading, lymphatic vessel invasion or venous invasion. Patients with N1 or N2 or UICC grade III more likely received systemic therapy than patients with lymph node involvement N0 or UICC grade IIB (Table 3).

**Table 3:** Distribution of tumor characteristics (gender, age at diagnosis, Charlson comorbidity index, diagnosis by ICD10 code, tumor size T, lymph node involvement N, stage UICC, grading, lymphatic vessel invasion, venous invasion) of stage IIB and III patients at initial diagnosis after primary systemic therapy

		Primary systemic therapy						
		No		Yes		Total		Chi-square
		*N*^5^	(%)	*N*^4^	(%)	*N*^4^	(%)	*p*
Gender	Female	12	38.7	10	35.7	22	37.3	0.812
	Male	19	61.3	18	64.3	37	62.7	
Diagnosis age	40.0 – 49.9	1	3.2	2	7.1	3	5.1	0.519
	50.0 – 59.9	5	16.1	9	32.1	14	23.7	
	60.0 – 69.9	8	25.8	7	25.0	15	25.4	
	70.0 – 79.9	14	45.2	8	28.6	22	37.3	
	80.0 – 89.9	3	9	2	7.1	5	8.5	
Charlson-Comorbidity-Index	0	17	54.8	14	50.0	31	52.5	0.701
	1	6	19.4	8	28.6	14	23.7	
	2 +	8	25.8	6	21.4	14	23.7	
Localization ICD10 Code	C17.0 Duodenum	22	71.0	12	42.9	34	57.6	0.091
	C17.1 Jejunum	7	22.6	12	42.9	19	32.2	
	C17.2 Ileum	2	6.5	4	14.3	6	10.2	
Tumor size T	T1/T2	2	6.5	2	7.1	4	6.8	0.689
	T3	6	19.4	8	28.6	14	23.7	
	T4	23	74.2	18	64.3	41	69.5	
Lymph node involvement	N0	14	45.2	3	10.7	17	28.8	0.013
	N1	12	38.7	16	57.1	28	47.5	
	N2	5	16.1	9	32.1	14	23.7	
Stadium UICC	IIB	14	45.2	3	10.7	17	28.8	0.013
	IIIA	12	38.7	16	57.1	28	47.5	
	IIIB	5	16.1	9	32.1	14	23.7	
Grading	G1/G2	21	67.7	14	50.0	35	59.3	0.166
	G3/G4	10	32.3	14	50.0	24	40.7	
Lymphatic vessel invasion	L0	13	41.9	7	25.0	20	33.9	0.073
	L1	12	38.7	19	67.9	31	52.5	
	LX/ns^6^	6	19.4	2	7.1	8	13.6	
Venous Invasion	V0	19	61.3	21	75.0	40	67.8	0.212
	V1/2	3	9.7	4	14.3	7	11.9	
	VX/ns^5^	9	29.0	3	10.7	12	20.3	
Total		31	100.0	28	100.0	59	100.0	

^5^Number of patients

^6^*Ns* not specified

#### Systemic therapy in patients with stage IV SBA

Of *n* = 58 patients with stage IV tumors at initial diagnosis, primary systemic therapy was given to *n* = 44 (75.9%) individuals (suppl. table 3). The systemic therapy in stage IV patients comprised folic acid and 5-fluorouracil, most frequently combined with oxaliplatin (FOLFOX, 36 patients), additionally with irinotecan (FOLFOXIRI, 5 patients), or irinotecan instead of oxaliplatin (FOLFIRI, 3 patients). One patient had hyperthermic intraperitoneal chemotherapy (HIPEC) for peritoneal carcinosis.

There was no significant difference between patients receiving systemic therapy and those who did not regarding sex, age at diagnosis, Charlson co-morbidity index, diagnosis by ICD10 code, tumor size T, lymph node involvement N, grading, lymphatic vessel invasion, venous invasion in the cohort analysed (suppl. table 4). Mean and median follow-up in stage IV patients was 3.9 (95% CI 3.1–4.7) and 4.9 (95% CI 4.1–5.8) years, respectively.

We observed that patients with primary systemic therapy had a higher 1-year survival rate (68.2%) compared to patients without primary systemic therapy (42.9%) with a median survival of 1.2 years with primary systemic therapy compared to 0.5 years without (*p* = 0.395, suppl. table 5). Multivariable cox-regression analysis resulted in a significant difference in survival depending on systemic therapy (HR 0.325, 95% CI 0.107–0.985, *p* = 0.047) and venous invasion (*p* = 0.038) (suppl. table 6).

**Fig. 3:**
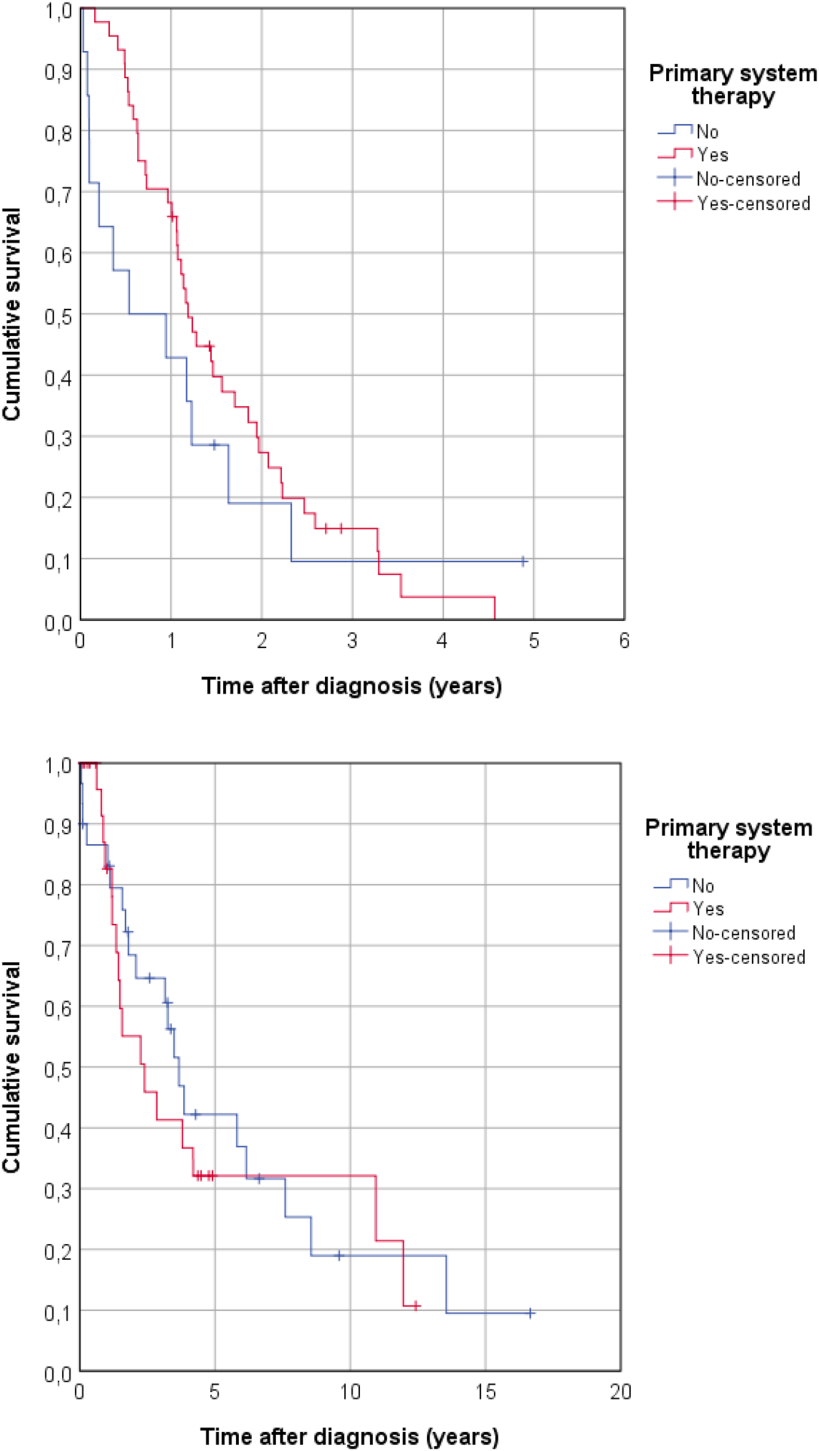
**A** Survival function (Kaplan–Meier curve) of stage IV patients at initial diagnosis after primary systemic therapy (yes/no), *p* = 0.306. **B** Survival function (Kaplan–Meier curve) of stage IIB/III patients at initial diagnosis after primary systemic therapy (Yes/No)

Of note, Kaplan–Meier analysis (Fig. 3a) also illustrated improved survival in the first years upon systemic therapy administration to patients diagnosed at stage IV. However, there was no significant difference in 5-year overall survival of both groups (*p* = 0.306).

#### Systemic therapy in patients with stage IIB and III small bowel cancer

The predominant chemotherapy in stage IIB and III patients was the combination of Folic Acid, 5-Fluorouracil and Oxaliplatin (FOLFOX, 17 patients), most frequently following the FOLFOX-4 scheme. One patient was offered a combination with Irinotecan (FOLFIRI). A Capecitabine-based therapy was offered to 8 patients, few patients received more uncommon protocols such as Mayo-scheme or Gemcitabine.

Mean and median follow-up in stage IIB/III patients was 8.0 (95% CI 5.6–10.4) and 4.9 years (95% CI 2.1–7.7), respectively. Subgroup analyses of other patient groups did not lead to a clear general difference in survival depending on chemotherapy application. Within the small subgroup of patients not receiving surgery, stage IIB and III patients being treated with systemic therapy showed an even worse outcome with a 3-year survival rate of 41.3% versus 64.6% of patients not receiving systemic therapy. This was complementary with a median survival of 2.4 (95% CI 0.5–4.3) years versus 3.7 years (95% CI 2.9–4.4, suppl. table 5). Also, Kaplan–Meier analysis revealed no significant difference in overall survival of patients diagnosed at stage IIB/III with regard to primary systemic therapy administration (*p* = 0.694, Fig. 3a, b). Multivariable cox-regression analysis resulted in a significantly worse hazard ratio of patients with primary systemic therapy of 2.673 (95% CI 1.056 – 6.771; *p* = 0.038) (suppl. table 6).

## Discussion

Small bowel adenocarcinoma (SBA) remains a rare malignancy accounting for less than 5% of all the gastrointestinal tract cancers (Hatzaras et al. 2007); (Sakae et al. 2017). As a consequence of the infrequent incidence of SBA, there is a lack of extensive clinical, aetiology and pathological information and it remains challenging to diagnose, treat, understand and prevent the disease. (Aparicio et al. 2014); (Farhat et al. 2008). Therefore, most treatment recommendations have been extrapolated from trials in colorectal cancer.

However, comparing overall survival of small intestine carcinomas with colon carcinoma for which treatment guidelines are well defined, we demonstrated a significantly lower 5-year survival rate and median survival for patients with small intestine carcinomas. Even though the numbers of patients with small bowel cancer in our analysis was small compared to the large cohort of patients with colon cancer, the design of our analysis as an area-wide, population-based, cross-sector analysis of all cancer patients and the logistic set up of receiving medical information from all regional pathologists and clinicians on diagnosis, treatment, and follow-up of those cancer patients mitigates the risk of selection bias in our cohort. As a result, subgroups of our analysis were generally well balanced. Particularly, investigating gender differences in epidemiology of small intestine cancers did not reveal any major differences.

Surgery was found to be the treatment of choice. The high rate of 84.6% of patients receiving surgery was surprising and presumably based on a high rate of patients with severe symptoms such as ileus, as at least 31.4% of our patients were staged as UICC stage IV cancers (potentially even more as in 18.8% of patients initial stage was not available). Although extensive surgery in stage IV metastatic colorectal cancer patients is certainly approached by many experts, it may also reflect the lack of reliable data on other treatment modalities particularly chemotherapy in patients with small intestine carcinomas. However, this arguably high surgery rate may also be due to SBA often presenting with a local complication such as bowel or gastric outlet obstruction and (occult) gastrointestinal bleeding obstruction. These complications require surgery irrespective of the overall oncologic concept and rationale (Dabaja et al. 2004); (Negoi et al. 2015; Gustafsson et al. 2008).

Evaluation of efficacy was obviously limited by small numbers in diverse stages of the disease. However, just as for colon cancer, in cancers with distant metastases the differences were significant and resulted in an increased median overall survival. Yet, the results were different for stage IIB and III patients where systemic therapy was not shown to exhibit any benefit. The reasons for that may be diverse. First and foremost, randomized, adequately large studies on drug efficacy in small intestine cancer are widely lacking. NCCN guidelines recommend FOLFOX, CAPEOX, or FOLFOXIRI (infusional 5-FU, LV, oxaliplatin, irinotecan) and potential combination with bevacizumab as first-line therapy for advanced disease in patients appropriate for intensive therapy (Benson et al. 2019). Recommendations for patients who are not appropriate for intensive therapy, 5-FU/LV or capecitabine (Benson et al. 2019). However, as also outlined in those guidelines these recommendations are only vaguely backed by small phase II trials. Prospective FOLFOX and CAPEOX phase II clinical trials reported an overall response rate (ORR) of 50% for CAPOX (Overman et al. 2009) and 48.5% for FOLFOX (Xiang et al. 2012). Additional data from an independent phase II study also reported an ORR of 45% for FOLFOX (Horimatsu et al. 2017). In contrast, the combination of 5-FU/doxorubicin/mitomycin C resulted in only 18% response rate (Gibson et al. 2005). Together, these data indicate that available chemotherapeutic regimens may be effective in SBA.

However, we were not able to observe a clear benefit from systemic chemotherapy in our SBA patients, which seemed somewhat in contrast to the well-established benefit in colon cancer. However, besides generally low numbers, we recorded the use of multiple diverse chemotherapeutic regimens such as FOLFOX, FOLFIRI, FOLFOXIRI and even HIPEC. This may mostly be due to a lack of evidence and large randomized trials for this tumor entity. The majority of the studies on SBA are either retrospective or case series investigating combinations of chemotherapeutics, mainly doublets of fluoropyrimidines associated with oxaliplatin or irinotecan. Novel agents, such as immunotherapy have mostly been investigated for small patient cohorts of 8–19 patients and mostly in basket trials further limiting the read out from these data (Nardo et al. 2022). Thus, SBA is generally still treated with chemotherapeutic agents commonly in use for metastatic colorectal cancer (mCRC), which clearly advocated for further collection of SBA data particularly on treatment outcome.

Furthermore, our analysis may contain some overlap between palliative and adjuvant treatment potentially when local surgery was necessary due to complications but not sufficient in terms of the overall oncological concept.

Finally, besides novel insights to treatment and survival of these patients, our analysis also clearly underlines that patients with SBA were diagnosed late, as more than 1/3 of all patients suffered from stage IV tumors at diagnosis. These findings are in line with a previous report that patients with SBA tend to present with a higher stage and grade compared with those with CRC. However, in contrast to this report we did not confirm that these patients were noticeably younger as the largest age subgroup was patients between 70 and 79.9 years.

Overall, our work furthermore adds to the perception that small bowel cancer is distinct from colon cancer in terms of diagnosis, prognosis, and therapeutic approach. It would, therefore, be desirable to have more data and expert insight available in guidelines for small bowel cancer. So far the only available comprehensive guidance comes from the French intergroup clinical practice guidelines for SBA (Locher et al. 2018) and the NCCN Clinical Practice Guidelines in Oncology (Benson et al. 2019).

## Conclusion

Our population-based characterization of SBA showed a distinct epidemiology compared to colon cancer, particularly as the 5-year survival rate was significantly lower. By far, most patients underwent primary surgery. Systemic therapy seemed to have an impact in UICC stage IV patients but not in UICC stage IIB or III. Given a tumor biology and epidemiology distinct from colon cancer, more reliable data and clinical studies particularly on systemic treatment are warranted, ultimately substantiating the few clinical guidelines available.

## Supplementary Information

Below is the link to the electronic supplementary material.

Supplementary file1 (DOCX 0 KB)
