# Supplementary material for: Characteristics and outcome of patients with small bowel adenocarcinoma (SBA)
Source: J Cancer Res Clin Oncol. 2022 Sep 26;149(8):4579–90. doi: 10.1007/s00432-022-04344-z (PMC10349691; doi:10.1007/s00432-022-04344-z)
Supplement: Supplementary file 1 — Supplementary file1 (DOCX 0 KB) [file 432_2022_4344_MOESM1_ESM.docx]

**Supplemental Material**

**Suppl. Figure 1**


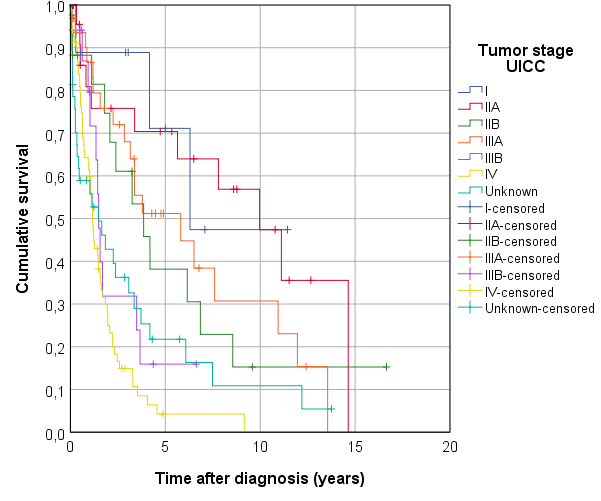


|  | | 5-year-survival rate | Median survival (years) |
| --- | --- | --- | --- |
| Stage UICC | I | 71,1% | 6,3 |
|  | IIA | 70,4% | 10,0 |
|  | IIB | 38,2% | 3,9 |
|  | IIIA | 51,2% | 5,8 |
|  | IIIB | 15,9% | 1,5 |
|  | IV | 4,3% | 1,2 |
|  | Unknown | 21,7% | 1,5 |

**Suppl. Figure 1:** Survival analysis (Kaplan-Meier curve) of patients with small bowel cancer depending on tumor stage at initial diagnosis.

**Suppl. Table 1**

|  | Stage UICC | I | IIA | IIB | IIIA | IIIB | IV | Unknown |
| --- | --- | --- | --- | --- | --- | --- | --- | --- |
|  |  | *p* | *p* | *p* | *p* | *p* | *p* | *p* |
| Log Rank (Mantel-Cox) | I |  | 0.915 | 0.139 | 0.329 | 0.024 | 0.000 | 0.012 |
|  | IIA | 0.915 |  | 0.101 | 0.151 | 0.009 | 0.000 | 0.001 |
|  | IIB | 0.139 | 0.101 |  | 0.663 | 0.105 | 0.001 | 0.107 |
|  | IIIA | 0.329 | 0.151 | 0.663 |  | 0.027 | 0.000 | 0.012 |
|  | IIIB | 0.024 | 0.009 | 0.105 | 0.027 |  | 0.168 | 0.808 |
|  | IV | 0.000 | 0.000 | 0.001 | 0.000 | 0.168 |  | 0.126 |
|  | Unknown | 0.012 | 0.001 | 0.107 | 0.012 | 0.808 | 0.126 |  |

**Suppl. Table 1:** Significance of differences in overall survival between patients with different tumor stages at the time of first diagnosis. (p-value (significance)).

**Suppl. Table 2**

|  | | Primary systemic therapy | | | | | | | | | |
| --- | --- | --- | --- | --- | --- | --- | --- | --- | --- | --- | --- |
|  |  | No | | Yes | | Planned | | Unknown | | Total | |
|  |  | N^[[1]](#footnote-1)^ | (%) | N^3^ | (%) | N^3^ | (%) | N^3^ | (%) | N^3^ | (%) |
| Stage UICC | I | 9 | 69.2 | 1 | 7.7 |  |  | 3 | 23.1 | 13 | 100.0 |
|  | IIA | 24 | 80.0 | 3 | 10.0 | 1 | 3.3% | 2 | 6.7 | 30 | 100.0 |
|  | IIB | 14 | 73.7 | 3 | 15.8 |  |  | 2 | 10.5 | 19 | 100.0 |
|  | IIIA | 12 | 37.5 | 18 | 56.3 | 1 | 3.1% | 1 | 3.1 | 32 | 100,0 |
|  | IIIB | 6 | 35.3 | 9 | 52.9 | 1 | 5.9% | 1 | 5.9 | 17 | 100.0 |
|  | IV | 14 | 20.0 | 45 | 64.3 | 7 | 10.0% | 4 | 5.7 | 70 | 100.0 |
|  | Unknown | 20 | 47.6 | 6 | 14.3 | 6 | 14.3% | 10 | 23.8 | 42 | 100.0 |
|  | Total | 99 | 44.4 | 85 | 38.1 | 16 | 7.2% | 23 | 10.3 | 223 | 100.0 |

**Suppl. Table 2:** Distribution of primary systemic therapy among UICC stages at initial diagnosis.

**Suppl. Table 3**

| Primary systemic therapy | Total number of patients | Number of events | 1-year survival rate | 2-year survival rate | Median survival in years |
| --- | --- | --- | --- | --- | --- |
| No | 14 | 12 | 42.9% | 19.0% | 0.5 |
| Yes | 44 | 40 | 68.2% | 27.3% | 1.2 |

**Suppl. Table 3:** 1-/2-year survival rate and median survival of stage IV patients at initial diagnosis after primary systemic therapy.

**Suppl. Table 4**

| Tumor properties | | Univariable Cox-Regression | | | | Multivariable Cox-Regression | | | |
| --- | --- | --- | --- | --- | --- | --- | --- | --- | --- |
|  |  | *p* | HR^^[[2]](#footnote-2)^^ | Lower 95%-CI^^[[3]](#footnote-3)^^ | Upper 95%-CI^9^ | *p* | HR^8^ | Lower 95%-CI^9^ | Upper 95%-CI^9^ |
| Primary Systemic Therapy | No |  | 1.000 |  |  |  | 1.000 |  |  |
|  | Yes | 0.397 | 0.754 | 0.393 | 1.448 | 0.047 | 0.325 | 0.107 | 0.985 |
| Gender | F |  | 1.000 |  |  |  | 1.000 |  |  |
|  | M | 0.831 | 0.941 | 0.537 | 1.649 | 0.497 | 1.333 | 0.582 | 3.053 |
| Diagnosis age | < 59,9 | 0.874 | 1.000 |  |  | 0.184 | 1.000 |  |  |
|  | 60,0 – 69,9 | 0.697 | 1.157 | 0.556 | 2.404 | 0.579 | 1.399 | 0.427 | 4.580 |
|  | ˃ 70,0 | 0.979 | 0.991 | 0.486 | 2.017 | 0.225 | 0.543 | 0.202 | 1.457 |
| Charlson-Comorbidity-Index | 0 | 0.322 | 1.000 |  |  | 0.664 | 1.000 |  |  |
|  | 1 | 0.720 | 0.888 | 0.463 | 1.702 | 0.983 | 1.106 | 0.404 | 2.429 |
|  | 2+ | 0.199 | 1.596 | 0.783 | 3.253 | 0.442 | 1.089 | 0.562 | 3.751 |
| Localization ICD10 - Code | C17.0 Duodenum | 0.325 | 1.000 |  |  | 0.935 | 1.000 |  |  |
|  | C17.1 Jejunum | 0.234 | 0.654 | 0.325 | 1.316 | 0.741 | 0.854 | 0.335 | 2.175 |
|  | C17.2 Ileum | 0.518 | 1.313 | 0.575 | 2.999 | 0.889 | 1.097 | 0.298 | 4.045 |
| Tumor size T | T1/T2 | 0.666 | 0.725 | 0.168 | 3.121 | 0.663 | 0.642 | 0.088 | 4.696 |
|  | T3 | 0.158 | 0.471 | 0.216 | 1.025 | 0.100 | 0.447 | 0.171 | 1.168 |
|  | T4 (Reference)^^[[4]](#footnote-4)^^ | 0.079 | 1.000 |  |  | 0.437 | 1.000 |  |  |
|  | TX/ns^10^ | 0.426 | 1.304 | 0.678 | 3.507 | 0.928 | 0.923 | 0.156 | 5.338 |
| Lymph node involvement | N0 | 0.097 | 1.000 |  |  | 0.419 | 1.000 |  |  |
|  | N1 | 0.201 | 1.787 | 0.735 | 4.349 | 0.189 | 2.387 | 0.651 | 8.751 |
|  | N2 | 0.820 | 0.885 | 0.309 | 2.533 | 0.975 | 0.979 | 0.258 | 3.708 |
|  | NX/ns^10^ | 0.063 | 2.317 | 0.955 | 5.621 | 0.472 | 2.134 | 0.271 | 16.787 |
| Grading | G1/G2 | 0.781 | 1.000 |  |  | 0.638 | 1.000 |  |  |
|  | G3/G4 | 0.832 | 1.066 | 0.591 | 1.924 | 0.352 | 0.706 | 0.340 | 1.468 |
|  | GX/ns^10^ | 0.482 | 1.387 | 0.557 | 3.458 | 0.905 | 0.930 | 0.283 | 3.054 |
| Lymph node invasion | L0 | 0.522 | 1.000 |  |  | 0.068 | 1.000 |  |  |
|  | L1 | 0.664 | 1.216 | 0.503 | 2.939 | 0.206 | 0.417 | 0.108 | 1.619 |
|  | LX/ns^10^ | 0.289 | 1.534 | 0.696 | 3.382 | 0.021 | 0.083 | 0.010 | 0.682 |
| Venous invasion | V0 | 0.147 | 1.000 |  |  | 0.038 | 1.000 |  |  |
|  | V1/V2 | 0.166 | 2.227 | 0.717 | 6.914 | 0.294 | 2.439 | 0.461 | 12.899 |
|  | VX/ns^10^ | 0.068 | 1.810 | 0.958 | 3.421 | 0.012 | 12.478 | 1.717 | 87.785 |

**Suppl. Table 4:** Comparison of univariable and multivariable Cox regression of tumor characteristics for overall survival in the evaluation population of stage IV patients.

**Suppl. Table 5**

| Primary Systemic Therapy | Total number of patients | Number of events | 1-year survival rate | 2-year survival rate | 3-year survival rate | Median survival in years |
| --- | --- | --- | --- | --- | --- | --- |
| No | 31 | 20 | 86.5% | 68.4% | 64.6% | 3.7 |
| Yes | 28 | 17 | 82.6% | 55.1% | 41.3% | 2.4 |

**Suppl. Table 5:** 1-/2-/3-year survival and median survival of stage IIB/III patients at initial diagnosis after primary systemic therapy.

**Suppl. Table 6**

| Tumor properties | | Univariable Cox-Regression | | | | Multivariable Cox-Regression | | | |
| --- | --- | --- | --- | --- | --- | --- | --- | --- | --- |
|  |  | *p* | HR^^[[5]](#footnote-5)^^ | Lower 95%-CI^^[[6]](#footnote-6)^^ | Upper 95%-CI^12^ | *p* | HR^11^ | Lower 95%-CI^12^ | Upper 95%-CI^12^ |
| Primary systemic therapy | No |  | 1.000 |  |  |  | 1,000 |  |  |
|  | Yes | 0.574 | 1.208 | 0.625 | 2.336 | 0.038 | 2.673 | 1.056 | 6.771 |
| Gender | F |  | 1.000 |  |  |  | 1.000 |  |  |
|  | M | 0.237 | 0.674 | 0.350 | 1.296 | 0.070 | 0.386 | 0.138 | 1.083 |
| Diagnosis age | 30.0 – 49.9 | 0.636 | 1.000 |  |  | 0.221 | 1.000 |  |  |
|  | 50.0 – 59.9 | 0.608 | 1.725 | 0.214 | 13.877 | 0.788 | 1.378 | 0.133 | 14.237 |
|  | 60.0 – 69.9 | 0.805 | 1.305 | 0.158 | 10.773 | 0.345 | 3.444 | 0.264 | 44.904 |
|  | 70.0 – 79.9 | 0.426 | 2.276 | 0.300 | 17.270 | 0.160 | 5.960 | 0.495 | 71.694 |
|  | 80.0 – 89.9 | 0.371 | 2.675 | 0.310 | 23.114 | 0.201 | 5.140 | 0.418 | 63.140 |
| Charlson-Comorbidity-Index | 0 | 0.154 | 1.000 |  |  | 0.811 | 1.000 |  |  |
|  | 1 | 0.240 | 1.604 | 0.729 | 3.529 | 0.825 | 1.172 | 0.287 | 4.778 |
|  | 2+ | 0.063 | 2.202 | 0.957 | 5.066 | 0.521 | 1.410 | 0.493 | 4.034 |
| Localization  ICD10 - Code | C17.0 Duodenum | 0.347 | 1.000 |  |  | 0.341 | 1.000 |  |  |
|  | C17.1 Jejunum | 0.216 | 0.627 | 0.299 | 1.314 | 0.178 | 0.443 | 0.135 | 1.453 |
|  | C17.2 Ileum | 0.352 | 0.502 | 0.118 | 2.140 | 0.569 | 0.596 | 0.100 | 3.544 |
| Tumor size | T1/T2 | 0.715 | 1.000 |  |  | 0.950 | 1.000 |  |  |
|  | T3 | 0.488 | 0.563 | 0.111 | 2.864 | 0.788 | 1.332 | 0.164 | 10.787 |
|  | T4 | 0.732 | 0.775 | 0.181 | 3.318 | 0.752 | 1.439 | 0.150 | 13.791 |
| Lymph node involvement N | N0 | 0.180 | 1.000 |  |  | 0.118 | 1.000 |  |  |
|  | N1 | 0.656 | 0.839 | 0.388 | 1.817 | 0.432 | 0.534 | 0.112 | 2.551 |
|  | N2 | 0.190 | 1.808 | 0.745 | 4.386 | 0.633 | 1.555 | 0.254 | 9.521 |
| Grading | G1/G2 |  | 1.000 |  |  |  | 1.000 |  |  |
|  | G3/G4 | 0.425 | 0.766 | 0.398 | 1.475 | 0.653 | 1.378 | 0.340 | 5.583 |
| Lymphatic vessel invasion | L0 | 0.445 | 1.000 |  |  | 0.164 | 1.000 |  |  |
|  | L1 | 0.341 | 0.696 | 0.330 | 1.467 | 0.065 | 0.263 | 0.064 | 1.087 |
|  | LX/ns^12^ | 0.232 | 0.513 | 0.171 | 1.533 | 0.370 | 0.313 | 0.025 | 3.966 |
| Venous invasion | V0 | 0.220 | 1.000 |  |  | 0.163 | 1.000 |  |  |
|  | V1 | 0.581 | 1.316 | 0.497 | 3.484 | 0.058 | 3.761 | 0.957 | 14.776 |
|  | VX/ns^12^ | 0.121 | 0.505 | 0.213 | 1.198 | 0.980 | 1.024 | 0.153 | 6.861 |

**Suppl. Table 6:** Comparison of univariable and multivariable Cox regression of tumor characteristics for overall survival in the stage IIB and III evaluation population.

1. Number of patients [↑](#footnote-ref-1)
2. HR = Hazard ratio; primary systemic therapy in multivariable Cox regression adjusted for tumor characteristics. [↑](#footnote-ref-2)
3. CI = Confidence interval, ns = not specified [↑](#footnote-ref-3)
4. The reference of tumor size T was moved from T1/T2 to T4 because the group size was too small. For all other tumor characteristics, the reference is in the first row. [↑](#footnote-ref-4)
5. HR = Hazard ratio; primary systemic therapy in multivariable Cox regression adjusted for tumor characteristics. [↑](#footnote-ref-5)
6. CI = Confidence interval, ns = not specified [↑](#footnote-ref-6)
